# Associations of 24-H Movement Behavior Composition with Estimated Cardiorespiratory Fitness in School-Aged Children: A Compositional Data Analysis

**DOI:** 10.3390/children13040553

**Published:** 2026-04-16

**Authors:** Andrés Godoy-Cumillaf, Josivaldo de Souza-Lima, Maribel Parra-Saldias, Daniel Duclos-Bastias, Claudio Farias-Valenzuela, Eugenio Merellano-Navarro, José Bruneau-Chávez

**Affiliations:** 1 Grupo de Investigación en Educación Física, Salud y Calidad de Vida (EFISAL), Facultad de Educación, Universidad Autónoma de Chile, Temuco 4780000, Chile; 2 Facultad de Educación y Ciencias Sociales, Instituto del Deporte y Bienestar, Universidad Andres Bello, Las Condes, Santiago 7550000, Chile; josivaldo.desouza@unab.cl; 3 Departamento de Educación Física, Deporte y Recreación, Universidad de Atacama, Copiapó 1530000, Chile; maribel.parra@uda.cl; 4 iGEO, Escuela de Educación Física, Facultad de Filosofía y Educación, Pontificia Universidad Católica de Valparaíso, Valparaíso 2340025, Chile; daniel.duclos@pucv.cl; 5 METIS Research Lab, Facultad de Negocios y Tecnología, Universidad Alfonso X el Sabio (UAX), 28691 Madrid, Spain; 6 Escuela de Ciencias de la Actividad Física, Universidad de Las Américas, Santiago 1530000, Chile; claudio.farias.valenzuela@udla.cl; 7 Department of Physical Activity Sciences, Faculty of Education Sciences, Universidad Catόlica del Maule, Talca 3530000, Chile; emerellano@ucm.cl; 8 Departamento de Educación Física, Deportes y Recreación, Universidad de la Frontera, Temuco 4811230, Chile; jose.bruneau@ufrontera.cl

**Keywords:** 24-h movement behaviors, compositional data analysis, isotemporal substitution, sleep, sedentary behavior, cardiorespiratory fitness, children, physical activity

## Abstract

**Highlights:**

**What are the main findings?**

• The 24-h movement behavior composition was significantly associated with estimated cardiorespiratory fitness in school-aged children.
• Reallocating time to MVPA, particularly from sedentary behavior or sleep, was associated with higher estimated cardiorespiratory fitness.

**What are the implications of the main findings?**

• Children’s cardiorespiratory fitness should be studied considering the full 24-h movement behavior composition rather than isolated behaviors.
• Interventions that increase MVPA within the daily time-use composition may help improve cardiorespiratory fitness in school-aged children.

**Abstract:**

**Background/Objectives:** This study aimed to examine the association between 24-h movement behavior composition and estimate cardiorespiratory fitness in school-aged children using compositional data analysis, and to model the theoretical differences in estimated cardiorespiratory fitness associated with isotemporal reallocations of time between movement behaviors. **Methods:** A cross-sectional study was conducted in 222 schoolchildren aged 8 to 12 years (mean age 9.94 ± 0.69 years), with most participants aged 10 years. Twenty-four-hour movement behaviors were assessed objectively using wrist-worn accelerometers, and cardiorespiratory fitness was estimated from the 20 m shuttle run test using the Léger equation. Daily time-use composition was analyzed using isometric log-ratio coordinates and adjusted linear regression models were fitted. Estimated differences in cardiorespiratory fitness associated with 30-min isotemporal reallocations between behaviors were then modeled. **Results:** The 24-h movement behavior composition was significantly associated with estimated cardiorespiratory fitness. In isotemporal models, reallocating 30 min from sedentary behavior to sleep was associated with the largest modeled difference in estimated cardiorespiratory fitness, whereas other reallocations showed smaller estimated differences depending on the behavior displaced. Age was positively associated with estimated cardiorespiratory fitness, while sex showed a limited association. Bivariate analyses revealed weak or inconsistent associations, supporting the value of the compositional approach for capturing the interdependent nature of daily time use. **Conclusions:** Twenty-four-hour movement behavior composition was associated with estimated cardiorespiratory fitness in school-aged children. These findings support the use of compositional approaches to examine sleep, sedentary behavior, and physical activity jointly. However, given the cross-sectional design and the modeled nature of the reallocations, the estimated differences should be interpreted cautiously and not as direct causal or physiological effects.

## 1. Introduction

Cardiorespiratory fitness is a key indicator of health in pediatric populations and is commonly estimated in field settings using standardized tests such as the 20-m shuttle run test [1,2]. Recent high-level evidence has consistently shown that higher cardiorespiratory fitness in children and adolescents is associated with more favorable cardiometabolic, musculoskeletal, and cognitive health profiles, whereas low cardiorespiratory fitness is linked to adverse health outcomes and greater future cardiovascular risk [3,4,5,6]. For this reason, cardiorespiratory fitness is widely regarded as an important marker of current and future health during childhood and adolescence [3,5].

In parallel, the 24-h movement behavior paradigm has gained prominence as a more integrative framework for understanding daily behaviors in youth. This approach considers sleep, sedentary behavior (SB), light-intensity physical activity (LIPA), and moderate-to-vigorous physical activity (MVPA) as codependent components of a finite 24-h day, meaning that time spent in one behavior necessarily displaces time spent in another [7,8]. Accordingly, these behaviors should not be examined in isolation. Compositional data analysis (CoDA) has been proposed as an appropriate analytical approach because it accounts for the relative and interdependent nature of daily time-use data [7,8,9]. In addition, CoDA-based isotemporal reallocation analyses allow the estimation of theoretical differences in health outcomes under hypothetical reallocations of time between movement behaviors [9,10,11,12].

Although a growing body of research suggests that more favorable 24-h movement behavior compositions are associated with better health indicators in children and adolescents [13,14,15,16,17,18,19,20,21,22], recent evidence synthesis indicates that the literature remains heterogeneous and that the most consistent benefits are observed for MVPA, whereas evidence for more specific behavioral balances and outcome-specific associations remains limited [23]. In particular, much of the available literature has focused on broader health and cardiometabolic outcomes, and studies specifically examining the relationship between 24-h movement behavior composition and cardiorespiratory fitness in school-aged children remain scarce [19,22,24]. Moreover, evidence from isotemporal reallocation analyses specifically targeting cardiorespiratory fitness in this population remains limited [11].

Therefore, the primary aim of this study was to examine the association between 24-h movement behavior composition and estimated cardiorespiratory fitness in school-aged children using compositional data analysis. The secondary aim was to model the theoretical differences in estimated cardiorespiratory fitness associated with isotemporal reallocations of time between sleep, sedentary behavior, light-intensity physical activity, and moderate-to-vigorous physical activity.

## 2. Materials and Methods

### 2.1. Study Design

This study used a cross-sectional observational design and was conducted in a school-aged pediatric population. It was designed to examine the association between 24-h movement behavior composition and estimated cardiorespiratory fitness in school-aged children using compositional data analysis, and to model the theoretical differences in estimated cardiorespiratory fitness associated with isotemporal reallocations of time between movement behaviors. The analytical sample comprised 222 participants aged 8 to 12 years, with a mean age of 9.94 years (SD 0.69). Although the observed age range extended to 12 years, the age distribution was strongly concentrated at 10 years. The database used for the analyses included information on age, sex, estimated cardiorespiratory fitness, and daily time spent in each movement behavior component. Because these behaviors form a closed 24-h composition, the study was framed within a compositional data analysis approach to model the relative distribution of time across movement behaviors and its association with estimated cardiorespiratory fitness. No a priori sample size or statistical power calculation was performed, as the study was based on an available dataset and the final analytical sample was defined according to data completeness and validity criteria.

### 2.2. Participants

Participants were eligible if they had available data on age, sex, estimated cardiorespiratory fitness, and all 24-h movement behavior components, namely sleep, sedentary behavior, light physical activity, and moderate-to-vigorous physical activity. To be included in the analytical sample, participants also had to present complete information for all variables entered in the statistical models. Participants were excluded if they had missing data in any study variable, incomplete accelerometry-derived movement behavior data, or values that did not allow construction of a complete 24-h composition. In addition, records containing zero values in any movement behavior component were excluded before the compositional analyses, because log-ratio transformations require strictly positive values. A total of 245 children were initially assessed for eligibility. Of these, 23 were excluded due to insufficient usage time, incorrect use, and/or technical failures. Accordingly, the final analytical sample consisted of 222 schoolchildren with complete valid data for the variables included in the present analyses. Of these, 130 were girls and 92 were boys. Participants ranged in age from 8 to 12 years; however, the age distribution was strongly concentrated at 10 years, which explains the mean age of 9.94 years despite the observed upper boundary of 12 years. Specifically, the sample included 15 participants aged 8 years, 7 aged 9 years, 183 aged 10 years, 10 aged 11 years, and 7 aged 12 years.

### 2.3. Procedure

Data collection was carried out using standardized procedures in a school-aged pediatric population to obtain information on age, sex, estimated cardiorespiratory fitness, and 24-h movement behaviors. For the present study, the movement behavior components considered were sleep, sedentary behavior, light physical activity, and moderate-to-vigorous physical activity. All collected data were incorporated into a unified database and reviewed for completeness and internal consistency before analysis. For accelerometry-derived movement behaviors, a valid day was defined as a complete 24-h recording. Sleep, sedentary behavior, light physical activity, and moderate-to-vigorous physical activity were derived from the same valid monitoring days to ensure construction of a complete daily composition. Only participants with complete and valid data for all study variables were retained in the analytical sample.

### 2.4. Ethical Considerations

The study was conducted in accordance with the ethical principles applicable to research involving human participants. Participation was voluntary, and all collected information was handled with strict confidentiality to protect participants’ identities.

Before data collection, parents or legal guardians were informed about the objectives and procedures of the study and provided written informed consent. In addition, the participation of the schoolchildren was based on their assent, according to their age and level of understanding.

The study protocol was reviewed and approved by the Ethics Committee of Universidad Autónoma de Chile (approval record No. CEC 31-22) and was carried out in accordance with current ethical standards for research involving human participants.

### 2.5. Variables and Instruments

#### 2.5.1. Cardiorespiratory Fitness

Cardiorespiratory fitness was assessed using the 20 m shuttle run test, which is widely used in pediatric populations and considered a valid and reliable measure of maximal aerobic fitness [1]. Based on test performance, estimated cardiorespiratory fitness (VO2max, mL/kg/min) was calculated using the equation proposed by Léger [2]. In the present study, estimated cardiorespiratory fitness was treated as the outcome variable.

#### 2.5.2. 24-H Movement Behaviors

Sedentary behavior, physical activity, and sleep were assessed using triaxial accelerometry with the GENEActiv device (Original Model, ActivInsights Ltd., Cambridgeshire, UK), which has shown validity for measuring these behaviors in pediatric populations. Participants wore the accelerometer on the non-dominant wrist for seven consecutive days. The device was configured at a sampling frequency of 100 Hz. Data were downloaded using GeneActiv software version 2.2 and processed using 30-s epochs. Daily time was classified into sedentary behavior, light physical activity, moderate physical activity, and vigorous physical activity according to the left-wrist cut-points proposed by Phillips et al. [25]. Specifically, the thresholds used were <87.5 for sedentary behavior, 87.5 to <250 for light physical activity, 250 to <750 for moderate physical activity, and ≥750 for vigorous physical activity. For the purposes of this study, moderate and vigorous physical activity were combined into a single moderate-to-vigorous physical activity variable.

Sleep duration was estimated using the Excel macro provided by ActivInsights, based on the Sadeh algorithm [26]. From this processing, daily time spent sleeping, showing sedentary behavior, performing light physical activity, and performing moderate-to-vigorous physical activity was obtained in minutes per day. All movement behavior components were derived from the same valid monitoring days, defined as days with complete 24-h accelerometer recording.

Because these behaviors represent interdependent parts of a daily 24-h composition, they were analyzed jointly under a compositional framework. To ensure comparability across participants, the observed times for each component were proportionally adjusted so that the daily sum equaled 1440 min. Records with zero values in any movement behavior component were excluded prior to compositional transformation.

#### 2.5.3. Covariables

Age was recorded in years, and sex was included as a sample characterization variable. Both variables were also considered adjustment covariates in the multivariable analyses.

### 2.6. Statistical Analysis

Statistical analyses were conducted using descriptive, bivariate, and compositional approaches. First, descriptive statistics were calculated for the total sample and stratified by sex, including sample size, mean, standard deviation, minimum, and maximum values for age, estimated cardiorespiratory fitness, and the 24-h movement behavior components. Differences between girls and boys were assessed using Welch’s *t* test for independent samples. Prior to inferential analyses, the distribution of continuous variables was assessed using the Shapiro–Wilk test and visual inspection of histograms and Q–Q plots. Based on these evaluations, parametric methods were considered appropriate. Bivariate associations between estimated cardiorespiratory fitness and the variables of interest were then examined using Pearson correlation coefficients, with the correlation coefficient and *p* value reported in each case.

The main analysis was conducted using compositional data analysis, acknowledging that time spent sleeping, showing sedentary behavior, performing light physical activity, and performing moderate-to-vigorous physical activity forms a closed 24-h composition. Movement behavior components were expressed in minutes per day and normalized to a total of 1440 min to standardize the daily composition across participants. Because the composition consisted of four parts, it was represented using three isometric log-ratio (ILR) coordinates derived from an orthonormal ILR basis defined through a sequential binary partition of the composition. Specifically, the first coordinate contrasted sleep with the remaining movement behaviors, the second contrasted sedentary behavior with the remaining waking activity components, and the third contrasted light physical activity with moderate-to-vigorous physical activity. Thus, the ILR transformation represented the relative structure and codependence of the daily movement behavior composition in real space for regression modeling.

Using the resulting three ILR coordinates, a multiple linear regression model was fitted with estimated cardiorespiratory fitness as the dependent variable. The model included the ILR coordinates as explanatory variables and was additionally adjusted for age and sex. To improve the interpretation and transparency of the analyses, measures of statistical uncertainty and model fit were reported, including regression coefficients, standard errors, 95% confidence intervals, *p* values, the coefficient of determination (R^2^), adjusted R^2^, and the residual standard deviation.

Finally, isotemporal reallocation analyses were performed based on the fitted compositional regression model to estimate the model-based differences in estimated cardiorespiratory fitness associated with hypothetical reallocations of 10, 15, and 30 min per day between movement behavior components while maintaining the total 24-h composition constant. These estimates were calculated relative to the sample mean composition normalized to 1440 min per day and should be interpreted as theoretical predictions derived from the fitted compositional model rather than as observed or causal effects.

All statistical analyses were performed using R software version 4.5.3. Statistical significance was set at *p* < 0.05.

## 3. Results

Table 1 summarizes the descriptive characteristics of the total sample and the sex-stratified groups. For age, estimated cardiorespiratory fitness, sleep time, sedentary behavior, light-intensity physical activity, and moderate-to-vigorous physical activity, the table reports sample size, mean, standard deviation, minimum, and maximum values. In the overall sample, the mean estimated cardiorespiratory fitness was 44.35. Within the 24-h movement behavior composition, sedentary behavior and sleep accounted for the greatest average daily time.

Table 2 presents the comparison between girls and boys for age, estimated cardiorespiratory fitness, and the movement behavior components. For each variable, mean, standard deviation, and the p value for the between-group comparison are reported. The mean values were similar in girls and boys across all variables analyzed. The *p* values ranged from 0.278 for age to 0.890 for sleep time.

Table 3 shows the bivariate correlations of estimated cardiorespiratory fitness with age, sleep time, sedentary behavior, light-intensity physical activity, and moderate-to-vigorous physical activity. For each association, the Pearson correlation coefficient and corresponding p value are reported. Among the bivariate associations, sleep time showed the largest positive correlation with estimated cardiorespiratory fitness (r = 0.2214; *p* = 0.0009), whereas light-intensity physical activity showed the largest negative correlation (r = −0.2737; *p* < 0.001). Correlations between estimated cardiorespiratory fitness and sedentary behavior, moderate-to-vigorous physical activity, and age were smaller in magnitude.

Table 4 presents the coefficients of the compositional regression model for estimated VO2 based on isometric log-ratio coordinates. Significant associations were observed for ilr1 and ilr3. Specifically, ilr1 was positively associated with estimated cardiorespiratory fitness (beta = 3.3567, SE = 1.0720, 95% CI = 1.2438 to 5.4697, *p* = 0.002), whereas ilr3 was inversely associated with estimated VO2 (beta = −2.2028, SE = 0.6614, 95% CI = −3.5064 to −0.8992, *p* = 0.001). In contrast, ilr2, age, and sex were not significantly associated with estimated cardiorespiratory fitness in the adjusted model. These results should be interpreted considering the overall model performance, which indicated that the model explained a modest proportion of the variance in estimated cardiorespiratory fitness.

Table 5 shows the fit indices of the compositional regression model for estimated cardiorespiratory fitness. The model included 222 participants and yielded an R^2^ of 0.1204 and an adjusted R^2^ of 0.1000, indicating that the explanatory variables accounted for a modest proportion of the variance in estimated cardiorespiratory fitness. The residual standard deviation was 3.6357. Accordingly, these findings should be interpreted with caution given the modest overall model fit.

Table 6 shows the model-based estimated differences in cardiorespiratory fitness associated with hypothetical isotemporal reallocations of 10, 15, and 30 min between movement behavior components. For each reallocation direction, the estimated absolute difference in cardiorespiratory fitness is presented relative to the mean composition standardized to 1440 min per day. The largest estimated positive differences were observed in reallocations from light-intensity physical activity to other behaviors, whereas the largest estimated negative differences were observed in reallocations from sleep or moderate-to-vigorous physical activity to light-intensity physical activity. For the 30-min reallocations, the estimated differences ranged from −1.07 to 1.45. These values represent theoretical predictions derived from the fitted compositional model and should not be interpreted as observed or causal effects.

**Figure 1:**
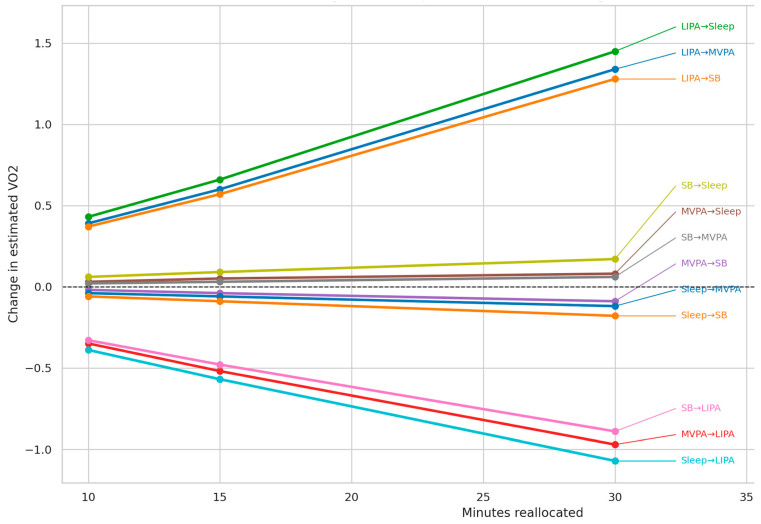
Predicted Changes in Estimated Cardiorespiratory Fitness According to Isotemporal Time Reallocations Within the 24-H Composition. VO2, estimated cardiorespiratory fitness; SB, sedentary behavior; LIPA, light-intensity physical activity; MVPA, moderate-to-vigorous physical activity.

Figure 1 illustrates the model-based estimated differences in cardiorespiratory fitness associated with hypothetical isotemporal reallocations of 10, 15, and 30 min between movement behaviors within the 24-h composition. The largest estimated positive differences were observed for reallocations from light-intensity physical activity to other behaviors, whereas the largest estimated negative differences were observed for reallocations toward light-intensity physical activity from moderate-to-vigorous physical activity or sleep. In general, the magnitude of the estimated differences increased with longer hypothetical reallocations, while the overall pattern remained consistent across scenarios. These estimates should be interpreted as theoretical outputs of the compositional model rather than as directly observed effects.

## 4. Discussion

This study examined the association between 24-h movement behavior composition and estimated cardiorespiratory fitness in school-aged children using compositional data analysis and modeled the theoretical differences in estimated cardiorespiratory fitness associated with isotemporal reallocations of time between movement behaviors. In this Chilean sample, the findings suggest that the relative distribution of time across sleep, sedentary behavior, light-intensity physical activity, and moderate-to-vigorous physical activity is associated with differences in estimated cardiorespiratory fitness, supporting the usefulness of the compositional approach for the integrated study of movement behaviors in pediatric populations [8,12,27].

The isotemporal reallocation analyses showed that some hypothetical reallocations involving sleep were associated with comparatively larger model-based estimated differences in cardiorespiratory fitness than other reallocation scenarios. These findings, however, should be interpreted with caution. Although sleep is an essential component of child health and has been linked to physiological recovery and favorable health indicators [13,14], the estimated differences observed in these simulations should not be interpreted as direct physiological improvements or causal effects. From a biological perspective, the larger estimated differences observed in some reallocations toward sleep are not straightforward to explain and may reflect, at least in part, mathematical properties of the compositional model rather than direct effects of that magnitude [8,12,27]. This is particularly relevant in analyses based on isometric log-ratio coordinates, in which small relative changes in one component of the composition may translate into amplified variation on the analytical scale because of the closed structure of the data [8]. In this context, the prominence of sleep in some simulations may have been influenced by the ILR transformation and the intrinsic codependence among the components of the day. Furthermore, the interpretation of isotemporal reallocations should take into account the baseline composition of the sample. Because the children included in this study accumulated a high proportion of time in sedentary behavior, the estimated differences associated with certain reallocations may have been conditioned by this initial movement pattern. Accordingly, these simulations should be interpreted as theoretical estimates derived from a specific reference composition rather than as literal physiological changes or directly generalizable effects in populations with different daily time-use distributions [8,12,27]. Therefore, the isotemporal reallocations presented in this study should be understood as theoretical compositional modeling scenarios rather than as direct predictions of real-world behavioral change. Accordingly, the magnitude of some estimated differences may appear amplified and should be interpreted with caution in terms of biological plausibility.

Beyond this caution, the findings also suggest that the distribution of movement behaviors across the day may be relevant to cardiorespiratory fitness in school-aged children. This interpretation is consistent with the conceptual basis of the 24-h movement behavior paradigm, which recognizes that sleep, sedentary behavior, and physical activity interact within a finite daily time budget [7,8]. It is also broadly consistent with previous compositional studies showing that more favorable movement behavior compositions, particularly those including more MVPA, tend to be associated with better health indicators in children and adolescents [17,18,19,19,20,21,22,23,24]. At the same time, the present findings should not be interpreted as evidence that sleep has a stronger physiological role than MVPA in relation to cardiorespiratory fitness. Rather, the results highlight the need to interpret compositional isotemporal models within their mathematical framework and considering the baseline time-use composition of the sample [8,12,27].

These findings should also be interpreted in relation to the broader literature on cardiorespiratory fitness in youth. Cardiorespiratory fitness is a multifactorial marker influenced by growth, maturation, habitual physical activity, and other biological and environmental factors [3,4,5,6]. In this context, it is plausible that the daily distribution of movement behaviors contributes to differences in estimated cardiorespiratory fitness, even though this relationship is unlikely to be explained by any single behavior in isolation. The weak or inconsistent associations observed in the bivariate analyses reinforce this point and support the value of compositional approaches for studying behaviors that are inherently codependent. From a practical perspective, these findings also suggest that considering the whole 24-h movement behavior composition may provide more informative insights than examining individual behaviors separately, although it remains important to avoid overinterpreting model-derived estimates as if they represented direct behavioral interventions or causal physiological responses.

The study has several strengths. It applies a compositional analytical framework that is conceptually appropriate for time-use data constrained within a 24-h day [7,8,9], and it extends this approach to a sample of Chilean schoolchildren, a population in which this methodology remains scarcely used [15,16]. In addition, by modeling isotemporal reallocations, the study provides a more integrative interpretation of how different hypothetical redistributions of time across movement behaviors may be associated with estimated cardiorespiratory fitness [7,8,9].

However, the findings should also be interpreted in light of several limitations. First, the cross-sectional design precludes establishing directionality or causality, and reverse causation cannot be ruled out; children with better cardiorespiratory fitness may also be more likely to accumulate more favorable movement behavior compositions. Second, although the compositional regression model indicated a significant association between the 24-h movement behavior composition and estimated cardiorespiratory fitness, the explained variance was modest, indicating that 24-h movement behavior composition represents only one of several factors associated with estimated cardiorespiratory fitness in children. Therefore, other biological, behavioral, and environmental factors not included in the analysis are also likely to contribute to cardiorespiratory fitness in children. Third, the estimates derived from the isotemporal simulations may overstate the magnitude of some model-based differences if interpreted outside the mathematical framework of compositional analysis. Because compositional data are constrained to a closed 24-h structure, and ILR coordinates express relative contrasts between behaviors, small relative differences may appear amplified on the analytical scale [8,12,27]. Fourth, although the main compositional models were adjusted for sex, the analyses were not fully stratified by sex; thus, possible sex-specific compositional patterns may not have been fully captured and should be explored in future studies [24]. Fifth, no a priori sample size or statistical power calculation was performed, as this study was based on an available dataset and the final analytical sample was determined according to data completeness and validity criteria. Accordingly, the findings should be interpreted considering the sample ultimately retained for analysis.

Future research should prioritize longitudinal designs, objective measurements, repeated assessments, and sex-stratified analyses to better understand how the redistribution of time across daily movement behaviors may influence cardiorespiratory fitness in children [17,24]. In addition, the use of compositional approaches in Latin America should continue to expand, as previous reviews have shown that much of the regional research on movement behaviors still relies on simpler analytical strategies [15,16].

## 5. Conclusions

In conclusion, 24-h movement behavior composition was associated with differences in estimated cardiorespiratory fitness in schoolchildren. These findings support the usefulness of compositional analysis for examining movement behaviors in an integrated manner in pediatric populations. However, due to the cross-sectional design of the study and the modeled nature of the isotemporal reallocations, the results should be interpreted with caution and should not be understood as evidence that any single behavior, including sleep, is more important than physical activity for cardiorespiratory fitness. Longitudinal and experimental studies are needed to confirm these associations and clarify their physiological and clinical relevance.

## Figures and Tables

**Table 1:** Descriptive characteristics of the study sample overall and stratified by sex.

Group	Variable	n	Mean	SD	Min	Max
Total	Age, years	222	9.94	0.69	8.0	12.0
Total	VO2	222	44.35	3.83	39.1	53.4
Total	Sleep, min/day	222	517.35	85.87	285.39	946.08
Total	SB, min/day	222	757.88	88.17	430.76	1070.9
Total	LIPA, min/day	222	84.66	24.3	19.9	147.5
Total	MVPA, min/day	222	80.11	34.18	14.65	179.22
GILs	Age, years	130	9.98	0.65	8.0	12.0
Girls	VO2	130	44.23	3.67	39.1	53.4
Girls	Sleep, min/day	130	516.68	87.51	285.39	946.08
Girls	SB, min/day	130	755.97	81.68	430.76	935.88
Girls	LIPA, min/day	130	86.15	23.74	19.9	147.5
Girls	MVPA, min/day	130	81.2	33.87	23.36	179.22
Boys	Age, years	92	9.88	0.74	8.0	12.0
Boys	VO2	92	44.52	4.06	39.1	53.4
Boys	Sleep, min/day	92	518.29	83.97	285.39	686.65
Boys	SB, min/day	92	760.58	97.0	590.79	1070.9
Boys	LIPA, min/day	92	82.56	25.05	19.9	122.35
Boys	MVPA, min/day	92	78.58	34.75	14.65	143.66

VO2, estimated cardiorespiratory fitness; SB, sedentary behavior; LIPA, light-intensity physical activity; MVPA, moderate-to-vigorous physical activity.

**Table 2:** Comparison of participant characteristics and movement behaviors between girls and boys.

	Girls Mean	Girls SD	Boys Mean	Boys SD	*p*-Value
Age, years	9.985	0.647	9.88	0.739	0.278
VO2	44.229	3.674	44.522	4.061	0.583
Sleep, min/day	516.68	87.508	518.286	83.968	0.890
SB, min/day	755.97	81.683	760.58	96.999	0.710
LIPA, min/day	86.149	23.735	82.556	25.046	0.283
MVPA, min/day	81.201	33.871	78.578	34.749	0.576

VO2, estimated cardiorespiratory fitness; SB, sedentary behavior; LIPA, light-intensity physical activity; MVPA, moderate-to-vigorous physical activity.

**Table 3:** Bivariate correlations between estimated cardiorespiratory fitness and age and movement behavior components.

Predictor	r	*p*-Value
Sleep	0.2214	<0.001
SB	−0.1254	0.062
LIPA	−0.2737	<0.001
MVPA	−0.038	0.573
Age	0.0594	0.378

SB, sedentary behavior; LIPA, light-intensity physical activity; MVPA, moderate-to-vigorous physical activity.

**Table 4:** Regression coefficients from the compositional data analysis model for estimated cardiorespiratory fitness.

Term	Beta	SE	95% CI Lower	95% CI Upper	*p*-Value
Intercept	34.2236	3.9174	26.5024	41.9448	<0.001
ilr1	3.3567	1.072	1.2438	5.4697	0.002
ilr2	1.0191	1.0081	−0.9678	3.0061	0.313
ilr3	−2.2028	0.6614	−3.5064	−0.8992	0.001
Age	0.5026	0.3591	−0.2052	1.2104	0.163
Male sex	0.1731	0.4989	−0.8102	1.1564	0.728

SE, Standard error; CI, confidence interval; ilr, isometric log-ratio.

**Table 5:** Model fit indices for the compositional regression model.

Metric	Value
Sample Size	222.0
R^2^	0.1204
Adjusted R^2^	0.1
Residual standard deviation	3.6357

**Table 6:** Predicted differences in estimated cardiorespiratory fitness associated with isotemporal reallocations of movement behaviors.

Reallocation	10 Min	15 Min	30 Min
LIPA to MVPA	0.39	0.6	1.34
LIPA to SB	0.37	0.57	1.28
LIPA to Sleep	0.43	0.66	1.45
MVPA to LIPA	−0.35	−0.52	−0.97
MVPA to SB	−0.02	−0.04	−0.09
MVPA to Sleep	0.03	0.05	0.08
SB to LIPA	−0.33	−0.48	−0.89
SB to MVPA	0.02	0.03	0.06
SB to Sleep	0.06	0.09	0.17
Sleep to LIPA	−0.39	−0.57	−1.07
Sleep to MVPA	−0.04	−0.06	−0.12
Sleep to SB	−0.06	−0.09	−0.18

SB, sedentary behavior; LIPA, light-intensity physical activity; MVPA, moderate-to-vigorous physical activity.

## Data Availability

The data presented in this study are available upon request from the corresponding author. The data are not publicly available due to ethical standards.

## Abbreviations

The following abbreviations are used in this manuscript:

BMI	Body mass index
CoDA	Compositional data analysis
CRF	Cardiorespiratory fitness
ilr	Isometric log-ratio
LPA	Light-intensity physical activity
LIPA	Light-intensity physical activity
MVPA	Moderate-to-vigorous physical activity
PA	Physical activity
SB	Sedentary behavior

## References

[1] Ruiz J.R., España V., Castro J., Artero E.G., Ortega F.B., García M., Jiménez D., Chillón P., Girela J., Mora J. (2011). Alpha-fitness test battery: Health-related field-based fitness tests assessment in children and adolescents. Nutr. Hosp..

[2] Léger L.A., Mercier D., Gadoury C., Lambert J. (1988). The multistage 20 metre shuttle run test for aerobic fitness. J. Sports Sci..

[3] Demchenko I., Prince S.A., Merucci K., Cadenas-Sanchez C., Chaput J.P., Fraser B.J., Manyanga T., McGrath R., Ortega F.B., Singh B. (2025). Cardiorespiratory fitness and health in children and adolescents: An overview of systematic reviews with meta-analyses representing over 125 000 observations covering 33 health-related outcomes. Br. J. Sports Med..

[4] Ortega F.B., Ruiz J.R., Castillo M.J., Sjöström M. (2008). Physical fitness in childhood and adolescence: A powerful marker of health. Int. J. Obes..

[5] Raghuveer G., Hartz J., Lubans D.R., Takken T., Wiltz J.L., Mietus-Snyder M., Perak A.M., Baker-Smith C., Pietris N., Edwards N.M. (2020). Cardiorespiratory Fitness in Youth: An Important Marker of Health: A Scientific Statement From the American Heart Association. Circulation.

[6] Chiang H.L., Chuang Y.F., Chen Y.A., Hsu C.T., Ho C.C., Hsu H.T., Sheu Y.H., Gau S.S., Liang L.L. (2024). Physical Fitness and Risk of Mental Disorders in Children and Adolescents. JAMA Pediatr..

[7] Dumuid D., Stanford T.E., Martin-Fernández J.-A., Pediši’c Ž., Maher C.A., Lewis L.K., Hron K., Katzmarzyk P.T., Chaput J.-P., Fogelholm M. (2018). Compositional Data Analysis for Physical Activity, Sedentary Time and Sleep Research. Stat. Methods Med. Res..

[8] Dumuid D., Pedišić Ž., Palarea-Albaladejo J., Martín-Fernández J.A., Hron K., Olds T. (2020). Compositional Data Analysis in Time-Use Epidemiology: What, Why, How. Int. J. Environ. Res. Public Health.

[9] Dumuid D., Pedišić Ž., Stanford T.E., Martín-Fernández J.A., Hron K., Maher C.A., Lewis L.K., Olds T. (2019). The compositional isotemporal substitution model: A method for estimating changes in a health outcome for reallocation of time between sleep, physical activity and sedentary behaviour. Stat. Methods Med. Res..

[10] Wang X., Zhang X., Yu J.J., Gao W., Wan M., Wen X. (2025). The Goldilocks Day for Preschoolers’ Health Outcomes: A Compositional Data Analysis of 24-H Movement Behaviors on Weekdays and Weekends. J. Exerc. Sci. Fit..

[11] Moore K.N., Castillo S., McAlister K.L., Chapman T.M., Crosley-Lyons R., Belcher B.R. (2025). Compositional data analysis of 24-hour movement behaviors as a predictor for cardiometabolic health outcomes among school-aged youth: A systematic review. J. Sports Sci..

[12] Clark C.C.T., Martins C.M.L. (2025). Twenty-Four-Hour Compositional Data Analysis in Healthcare: Clinical Potential and Future Directions. Int. J. Environ. Res. Public Health.

[13] Hossian M., Mielke G.I., Nisar M., Tremblay M.S., Khan A. (2025). Global research on 24-hour movement behaviours guidelines in children and adolescents: A systematic review. Int. J. Behav. Nutr. Phys. Act..

[14] Canadian Society for Exercise Physiology (2016). Canadian 24-Hour Movement Guidelines for the Children and Youth (5–17 Years): An Integration of Physical Activity, Sedentary Behaviour, and Sleep.

[15] Zhao H., Wu N., Haapala E.A., Gao Y. (2024). Association between meeting 24-h movement guidelines and health in children and adolescents aged 5–17 years: A systematic review and meta-analysis. Front. Public Health.

[16] Silva A.F.D., Martins P.C., Santiago L.N., Silva D.A.S. (2025). Mapping Evidence on Integrated 24-Hour Movement Behaviors in Children and Adolescents: A Scoping Review of Reviews. Children.

[17] Song H., Zhang B., Lu N., Liu Y., Lau P.W.C. (2025). Longitudinal associations between 24-hour movement behaviors and physical fitness in preschoolers: A compositional isotemporal substitution analysis. Sci. Rep..

[18] Song H., Lau P.W.C., Wang J., Liu Y., Song Y., Shi L. (2024). 24-H movement behaviors and physical fitness in preschoolers: A compositional and isotemporal reallocation analysis. J. Exerc. Sci. Fit..

[19] Reis L.N., Reuter C.P., Bergmann G.G., Mota J., Gaya A.C.A., Bandeira P.F., Schneiders L.D.B., Fochesatto C.F., Brand C., Gaya A.R. (2025). 24-hour movement components, cardiorespiratory fitness and cardiometabolic risk in children: A network perspective. Int. J. Environ. Health Res..

[20] Chung H.J., Kim H., Ma J., Chua T., Low S.T., Li D., Guo H., Chia M.Y.H. (2025). Associations between 24-hour movement guidelines and health-related quality of life among urban preschool children in Singapore, Japan, and China. Health Qual. Life Outcomes.

[21] Marshall Z.A., Runacres A., Hallal P.C., Jago R., Kwon S., Northstone K., Pate R.R., Puder J., Reilly J.J., Sardinha L.B. (2025). Associations between 24-hour movement compositions and cardiometabolic health in children and adolescents: A five-part compositional analysis using data from the International Children’s Accelerometery Database (ICAD). BMJ Open Sport Exerc. Med..

[22] Lu Z., Guo J., Liu C., Wu J., Zhao C., Wang F., Bao Y., Zhang H., Qi B., Li X. (2024). Reallocation of time to moderate-to-vigorous physical activity and estimated changes in physical fitness among preschoolers: A compositional data analysis. BMC Public Health.

[23] Kuzik N., Duncan M.J., Beshara N., MacDonald M., Silva D.A.S., Tremblay M.S. (2025). A systematic review and meta-analysis of the first decade of compositional data analyses of 24-hour movement behaviours, health, and well-being in school-aged children. J. Act. Sedentary Sleep Behav..

[24] Ricardo-Sejin J.R., Arango-Paternina C.M., Florez-Pregonero A., Patino-Villada F.A. (2025). Research characteristics about the 24-h movement behaviors in children and adolescents: A scoping review. BMC Public Health.

[25] Phillips L., Parfitt G., Rowlands A. (2013). Calibration of the GENEA accelerometer for assessment of physical activity intensity in children. J. Sci. Med. Sport.

[26] Sadeh A., Sharkey K.M., Carskadon M.A. (1994). Activity-based sleep-wake identification: An empirical test of methodological issues. Sleep.

[27] Sunda M., Gilic B., Sekulic D., Matic R., Drid P., Alexe D.I., Cucui G.G., Lupu G.S. (2022). Out-of-School Sports Participation Is Positively Associated with Physical Literacy, but What about Physical Education? A Cross-Sectional Gender-Stratified Analysis during the COVID-19 Pandemic among High-School Adolescents. Children.

